# Certain *Listeria monocytogenes* plasmids contribute to increased UVC ultraviolet light stress

**DOI:** 10.1093/femsle/fnab123

**Published:** 2021-09-09

**Authors:** Justin M Anast, Stephan Schmitz-Esser

**Affiliations:** Department of Animal Science, Iowa State University, Ames, IA 50011, USA; Interdepartmental Microbiology Graduate Program, Iowa State University, Ames, IA 50011, USA; Department of Animal Science, Iowa State University, Ames, IA 50011, USA; Interdepartmental Microbiology Graduate Program, Iowa State University, Ames, IA 50011, USA

**Keywords:** *Listeria monocytogenes*, foodborne illness, plasmid, ultraviolet light, food production stress, persistence

## Abstract

*Listeria monocytogenes* is the causative agent of the highly fatal foodborne disease listeriosis and can persist in food production environments. Recent research highlights the involvement of *L. monocytogenes* plasmids in different stress response mechanisms, which contribute to its survival in food production facilities. Ultraviolet (UV) light in the UVC spectrum (200–280 nm) is used in food production to control microbial contamination. Although plasmid-encoded UV resistance mechanisms have been described in other bacteria, no research indicates that *L. monocytogenes* plasmids contribute to the UV stress response. The plasmids of *L. monocytogenes* strains 6179, 4KSM and R479a are genetically distinct and were utilized to study the roles of plasmids in the UV response. Wild-type and plasmid-cured variant cells were grown to logarithmic or late-stationary phase, plated on agar plates and exposed to UVC for 60 or 90 s, and colony-forming units (CFUs) were determined. CFUs of 6179 and 4KSM, bearing pLM6179 and p4KSM, respectively, were significantly (*P*-value < 0.05) higher than those of the plasmid-cured strains in both logarithmic and stationary phases. No difference in survival was observed for the R479a strain. Our data show for the first time that certain *L. monocytogenes* plasmids contribute to the survival of UVC light stress.

## Teaser

Plasmids of the foodborne pathogen *Listeria monocytogenes* contribute to increased tolerance toward ultraviolet light.

## INTRODUCTION

The consumption of food products contaminated by foodborne pathogens poses a significant risk to public health (Scallan *et al*. 2011). Food producers attempt to mitigate the occurrences of outbreaks by combining multiple means of controlling microbial pathogens through a practice known as the ‘hurdle approach’. Frequently, good management practices can be individually insufficient, but when used in tandem with other practices, they significantly reduce the risk of contaminated food products. Ideally, the proper combination of hurdles used in food production and processing ensures that pathogens are either eliminated or prevented from reaching harmful levels, providing confidence that the final product will be safe for consumption (Mogren *et al*. 2018). One such hurdle commonly used in food production environments (FPEs) is short-wave ultraviolet light (UVC), referring to light demarcated between 200 and 280 nm on the electromagnetic spectrum (Gomez-Lopez *et al*. 2007). UVC may mediate bacterial inactivation through several mechanisms, including damage to bacteria in the form of pyrimidine dimers and the loss of cytoplasmic contents post-light absorption (Gomez-Lopez *et al*. 2007; Rastogi *et al*. 2010). UVC equipment is economically feasible, relatively easy to use and effective against most foodborne pathogens on flat, hard surfaces. The effectivity of UVC for inhibiting microbial growth has been extensively studied in several pathogens, including *Listeria monocytogenes* (Ozer and Demirci 2006; Keklik, Demirci and Puri 2009; Bernbom, Vogel and Gram 2011; Adhikari *et al*. 2015; Gayan *et al*. 2015).

*Listeria monocytogenes* is a Gram-positive facultative anaerobe that is the causative agent of listeriosis, a rare but highly fatal foodborne illness with mortality rates reaching as high as 30% (Ferreira *et al*. 2014; Buchanan *et al*. 2017). Additionally, *L. monocytogenes* is of considerable concern for food producers due to its persistence (i.e. long-term survival) in FPEs (Carpentier and Cerf 2011). The remarkable persistence of *L. monocytogenes* is due in part to a complex array of molecular mechanisms that provide increased protection against low pH (Cotter, Gahan and Hill 2000; Feehily *et al*. 2014; Lund, Tramonti and De Biase 2014), high osmotic pressure (Bucur *et al*. 2018), oxidative stress (Harter *et al*. 2017), disinfectants (Elhanafi, Dutta and Kathariou 2010; Muller *et al*. 2013, 2014), UVC (Kim *et al*. 2006) and temperatures as low as –0.4°C (Chan and Wiedmann 2009). In addition to chromosomally encoded stress response systems, it has been revealed that *L. monocytogenes* plasmids contribute to stress tolerance. Indeed, *L. monocytogenes* plasmids encode several putative stress response genes (Kuenne *et al*. 2010; Pontinen *et al*. 2017; Hingston *et al*. 2019; Naditz *et al*. 2019), and transcriptomic studies have demonstrated that *L. monocytogenes* plasmid genes were upregulated during oxidative, acidic and osmotic stress exposure (Hingston *et al*. 2019; Cortes *et al*. 2020). Furthermore, it has been shown that *L. monocytogenes* plasmids contribute to various stress conditions found in food production, such as increased temperature, high salt concentrations, lactic acid and disinfectants (Elhanafi, Dutta and Kathariou 2010; Pontinen *et al*. 2017; Naditz *et al*. 2019). Among the predicted *L. monocytogenes* plasmid-encoded stress response genes is a putative *uvrX* gene (Kuenne *et al*. 2010), annotated as being involved in UV stress response. *uvrX* genes are a part of the Y-family DNA polymerase and were initially identified in a prophage from the genome of *Bacillus subtilis* (Kunst *et al*. 1997; Sung *et al*. 2003; Timinskas and Venclovas 2019). UV tolerance has been investigated in *L. monocytogenes* (Ozer and Demirci 2006; Keklik, Demirci and Puri 2009; Bernbom, Vogel and Gram 2011; Adhikari *et al*. 2015; Gayan *et al*. 2015; Uesugi *et al*. 2016; Green *et al*. 2018), and the chromosomally encoded gene *uvrA* was shown to be necessary for UV stress survival (Kim *et al*. 2006). However, to the best of our knowledge, there are no studies in the current literature that demonstrate that *L. monocytogenes* plasmids are functionally involved in UV stress tolerance. The fact that plasmids are linked to UV tolerance in other organisms, including the *Pseudomonas* plasmid genes *rulAB* that encode proteins that assist in DNA repair (Sundin *et al*. 1996; Cazorla *et al*. 2008; Stockwell *et al*. 2013), raises the possibility that *L. monocytogenes* plasmids may also be involved in the UV stress response. Adding to this evidence, we have shown in our previous studies (Naditz *et al*. 2019; Cortes *et al*. 2020) that the plasmids of the persistent *L. monocytogenes* strains 6179, 4KSM and R479a are involved in different FPE-associated stress conditions. Interestingly, although the plasmids of each strain are different (Naditz *et al*. 2019), all three harbor a putative *uvrX* gene. Therefore, we sought to determine whether these three *L. monocytogenes* plasmids are involved in the survival of UV stress.

## METHODS

### Strains and maintenance

The selected plasmid-harboring *L. monocytogenes* strains 6179, R479a and 4KSM have previously been determined to be persistent in FPEs (Table 1) and represent three *L. monocytogenes* sequence types (ST121, ST8 and ST5). These strains have also been used extensively as models for studying *L. monocytogenes* stress response mechanisms (Muller *et al*. 2013, 2014; Casey *et al*. 2014; Fagerlund *et al*. 2016; Fox *et al*. 2016; Harter *et al*. 2017; Rychli *et al*. 2017; Muhterem-Uyar *et al*. 2018; Naditz *et al*. 2019; Cortes *et al*. 2020). Previously, these strains were cured of their plasmids to generate the corresponding plasmid-cured strains (Naditz *et al*. 2019). Wild-type (WT) and plasmid-cured strains were propagated from −80°C stock cultures and routinely maintained on tryptic soy agar (TSA; BD, Sparks, MD, USA) at 37°C and stored at 4°C. Although the average nucleotide identities shared between pLM6179 and pLMR479a were above 95%, overlap does not exceed 52% coverage (Naditz *et al*. 2019), demonstrating that the genetic contents of the three plasmids are highly distinct.

**Figure 1.**
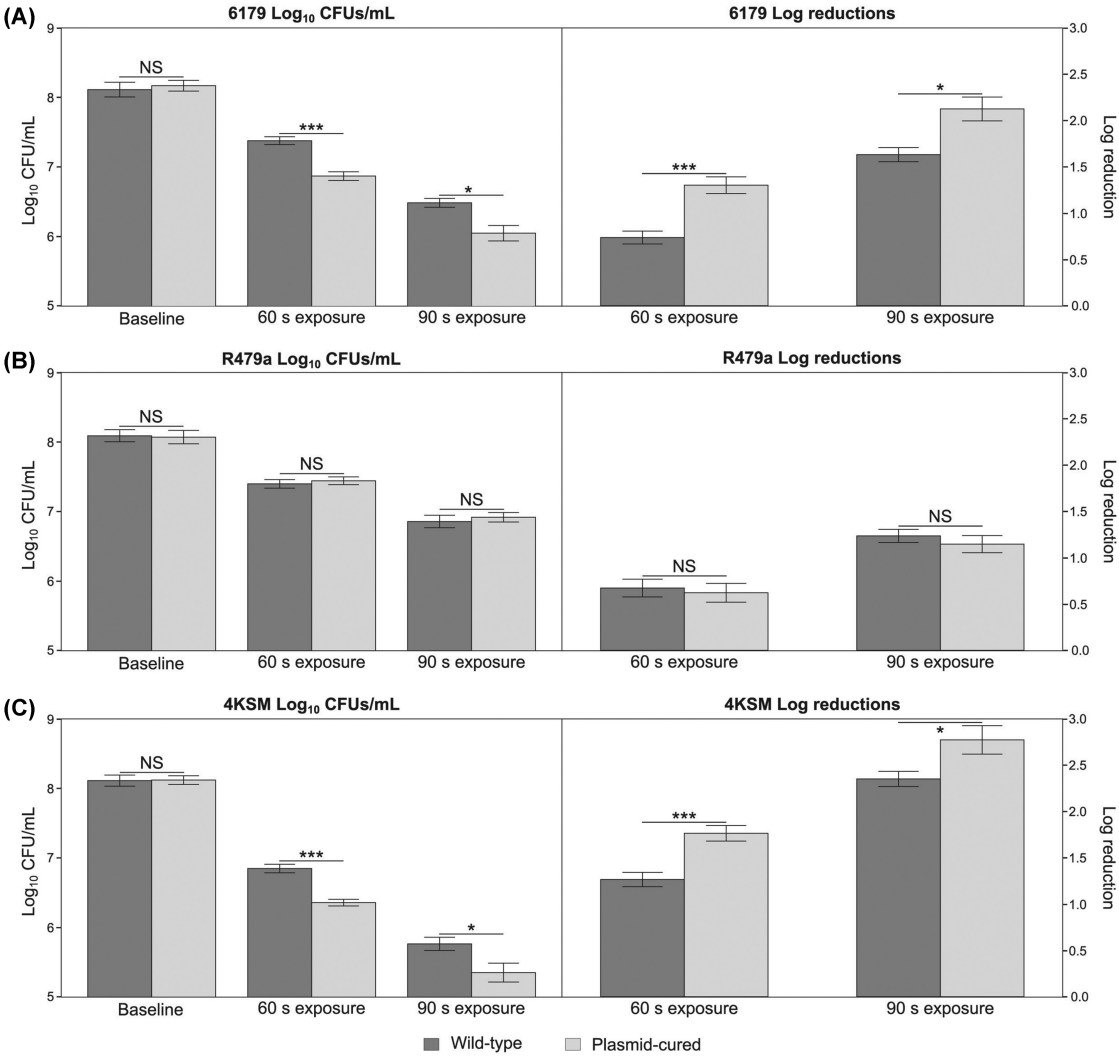
Survival of logarithmic phase WT and plasmid-cured *L. monocytogenes* strains under UVC stress at 20°C, displayed as log_10_ CFU/mL values (left panels) and log_10_ reduction values based on CFU/mL (right panels). Error bars show the standard error among four replicates. Strains appear as follows: **(A)** 6179; **(B)** R479a; **(C)** 4KSM. The baseline treatment refers to the standardized overnight (16 h) cultures that were never exposed to UVC. The 60 and 90 s treatments refer to the UVC-exposed cells. These cells were plated from the same standardized overnight as the baseline. *P*-values are as follows: *P ≤ 0.05, ***P ≤ 0.001; NS = not significant.

**Table 1.** Plasmid-harboring *L. monocytogenes* strains used in this study.

Strain	Sequence type	Country of origin	Isolation source, year	Plasmid	Plasmid size (kbp)	GenBank plasmid contig accession number	Reference
6179	ST121	Ireland	Cheese, 2000	pLM6179	62.2	HG813250	Schmitz-Esser *et al*. (2015a)
R479a	ST8	Denmark	Smoked salmon, 1996	pLMR479a	86.6	HG813248	Schmitz-Esser, Gram and Wagner (2015b)
4KSM	ST5	Austria	FPE, 2011	p4KSM	90.5	JYOJ01000032.1; JYOJ01000033.1^a^	Muhterem-Uyar *et al*. (2018)

a p4KSM consists of two contigs because the sequence has not been completed.

### UV light treatments and enumeration of CFUs

For all isogenic pairs, 5 mL of tryptic soy broth (TSB; BD, Sparks, MD, USA) was inoculated with a single colony and then incubated at 20°C with shaking at 200 rpm. Although many studies that utilize *L. monocytogenes* conduct overnight incubations at 37°C often due to its pathogenic life cycle, we decided to conduct cultivations in this study at 20°C to simulate temperatures found in FPEs more accurately. Cultures were then cultivated to either the logarithmic phase (16 h; optical density [OD] of ∼1.2) or late-stationary phase (48 h; OD of ∼2.1). These overnight cultures were then adjusted to an OD of 0.1 measured at 600 nm in phosphate-buffered saline (PBS). After the standardization of overnight concentrations, the WT strains and the corresponding plasmid-cured strains were serially diluted in PBS in 10-fold increments. Next, 100 µL of serially diluted culture from each strain was plated on TSA in triplicate to determine the colony-forming units (CFUs) in the starting culture as a baseline. For the UV treatment, 100 µL of cells from the same serial dilution as the baseline were spread-plated until dry in triplicate at a 10^–4^ dilution. The plate lids were removed, and UV stress exposure was conducted under aseptic conditions using a Spectroline^®^ Model ENF-240C UV lamp (Westbury, NY, USA) set to the short-wave UV function (254 nm) at a distance of 23 cm from each plate. UVC fluence rate was determined to be 30 µW/cm^2^ by measurement with a Spectroline^®^ Model DM-254XA short-wave UV meter, for total UVC dosages of 18 J/m^2^ in the 60 s exposure and 27 J/m^2^ in the 90 s exposure. Exposure durations of 60 and 90 s were applied for both logarithmic and late-stationary phase cells. After UVC exposure, plates were sealed and incubated at 37°C for 24 h to determine CFUs. Each experiment was performed in four independent biological replicates, with each replicate conducted on separate days. Log_10_ reductions were calculated from the obtained CFU counts after exposure to UVC using the following formula: log_10_ reduction = log_10_ (baseline CFUs) − log_10_ (UVC exposed CFUs). The decimal reduction time (*D*-value) was determined by plotting log_10_ CFU/mL as the *y*-axis and time of exposure as the *x*-axis and using the negative reciprocal of the slopes of regression lines.

### Sequence analysis

Annotation of plasmid genes and comparative Basic Local Alignment Search Tool (BLAST) searches were conducted in the Pathosystems Resource Integration Center (PATRIC) (Wattam *et al*. 2017). To further elucidate the putative function of annotated plasmid proteins, amino acid sequences were used as a query to search for putative functional protein domain homologs using the Protein Family database (PFAM) (Mistry *et al*. 2021). Protein homologs between the plasmids were determined using a local BLAST+ (2.11.0) database with 90% amino acid identities and an *e*-value of 0.01 as thresholds.

### Statistical analysis

A Student's unpaired, two-tailed *t*-test (α = 0.05) was conducted in Microsoft Excel 2016 to compare differences in population means before and after exposure to UV light. JMP Pro 14 was used for graphing and determination of slopes and standard errors.

## RESULTS

### CFUs of WT strains compared with plasmid-cured strains before UV stress

This study seeks to elucidate the contribution of *L. monocytogenes* plasmids in the survival of cells after UVC light exposure. Therefore, it is vital to assess whether removing the native plasmid caused a phenotype in planktonically grown cells. To ensure that any differences in survival between the WT and plasmid-cured strains were due to UV stress and not due to plasmid curing, the standardized overnight cultures were serially diluted on TSA and incubated without exposure to UVC. After both 16 and 48 h of growth in TSB, no significant differences (*P*-value > 0.05) in CFUs were observed between each strain and its plasmid-cured counterpart, demonstrating that plasmid curing did not affect cell survival under controlled nonstress conditions.

### Survival of WT strains compared with plasmid-cured strains after UVC radiation exposure

This study aimed to analyze and compare the UVC stress survival phenotypes between *L. monocytogenes* WT and plasmid-cured strains. Because the growth phase can influence the stress response systems of *L. monocytogenes* (Utratna *et al*. 2014), we analyzed the strains at two different growth phases. We first sought to investigate the effect of plasmids on UVC stress survival in cells grown to the logarithmic phase before UVC exposure. WT strains of *L. monocytogenes* 6179 and 4KSM that harbored their respective native plasmid survived significantly better than their plasmid-cured isogenic pairs after 60 s (log_10_ CFU and log_10_ reductions *P-*value < 0.001) and 90 s (log_10_ CFU and log_10_ reductions *P-*value < 0.05) UVC exposure times (Table 2; Fig. 1A and C). In contrast, no significant difference in CFUs or log_10_ reductions between the UVC tolerance of the R479a WT and plasmid-cured strains was observed at either the 60 or 90 s exposure time (Table 2; Fig. 1B). As expected, for all strains, the log_10_ reduction values were higher for the 90 s UVC exposure compared with the 60s UVC exposure. The degree of *L. monocytogenes* inactivation was measured at three separate UVC dosages (i.e. length of exposure) for the purpose of determining the inactivation rate of strains in response to UVC, as opposed to merely quantifying UVC sensitivity at a single dose. It should be noted that this study is limited by the low number of dosages (*n *= 3) used to produce a linear fit for calculation of *D*-values. We found that in logarithmic phase cells, the *D*-value of *L. monocytogenes* strains was reduced when the plasmids pLM6179 and pLMR479a, respectively, were absent. In contrast, the *D*-value of *L. monocytogenes* R479a slightly increased (although nearly identical) when pLMR479 was removed (Table 2). These *D*-value data indicate that the time to reduce the number of *L. monocytogenes* cells of strains 6179 and 4KSM by one log is shortened by removing the plasmid. In the case of R479a, the removal of the plasmid resulted in a slight increase in time needed to reduce *L. monocytogenes* R479a by one log.

**Table 2.** *Listeria monocytogenes* log_10_ reductions and calculated *D*-values after UVC exposure.

Strain	Logarithmic growth phase log_10_ reduction 60 s exposure^a^	Logarithmic growth phase log_10_ reduction 90 s exposure^a^	Stationary growth phase log_10_ reduction 60 s exposure^a^	Stationary growth phase log_10_ reduction 90 s exposure^a^	Logarithmic growth phase *D*-value (min)	Stationary growth phase *D*-value (min)
6179	0.73 ± 0.07	1.63 ± 0.07	0.86 ± 0.05	1.61 ± 0.09	0.97	1.03
6179-cured	1.30 ± 0.09	2.12 ± 0.12	1.24 ± 0.06	2.16 ± 0.06	0.72	0.74
R479a	0.67 ± 0.09	1.23 ± 0.07	0.49 ± 0.05	0.99 ± 0.06	1.24	1.82
R479a-cured	0.62 ± 0.10	1.14 ± 0.09	0.60 ± 0.07	0.94 ± 0.03	1.33	1.77
4KSM	1.26 ± 0.07	2.35 ± 0.08	0.76 ± 0.06	1.47 ± 0.04	0.66	1.16
4KSM-cured	1.76 ± 0.08	2.77 ± 0.15	1.02 ± 0.05	1.95 ± 0.05	0.55	0.85

a Values are reported as the means of the log_10_ reductions ± the standard error.

**Figure 2.**
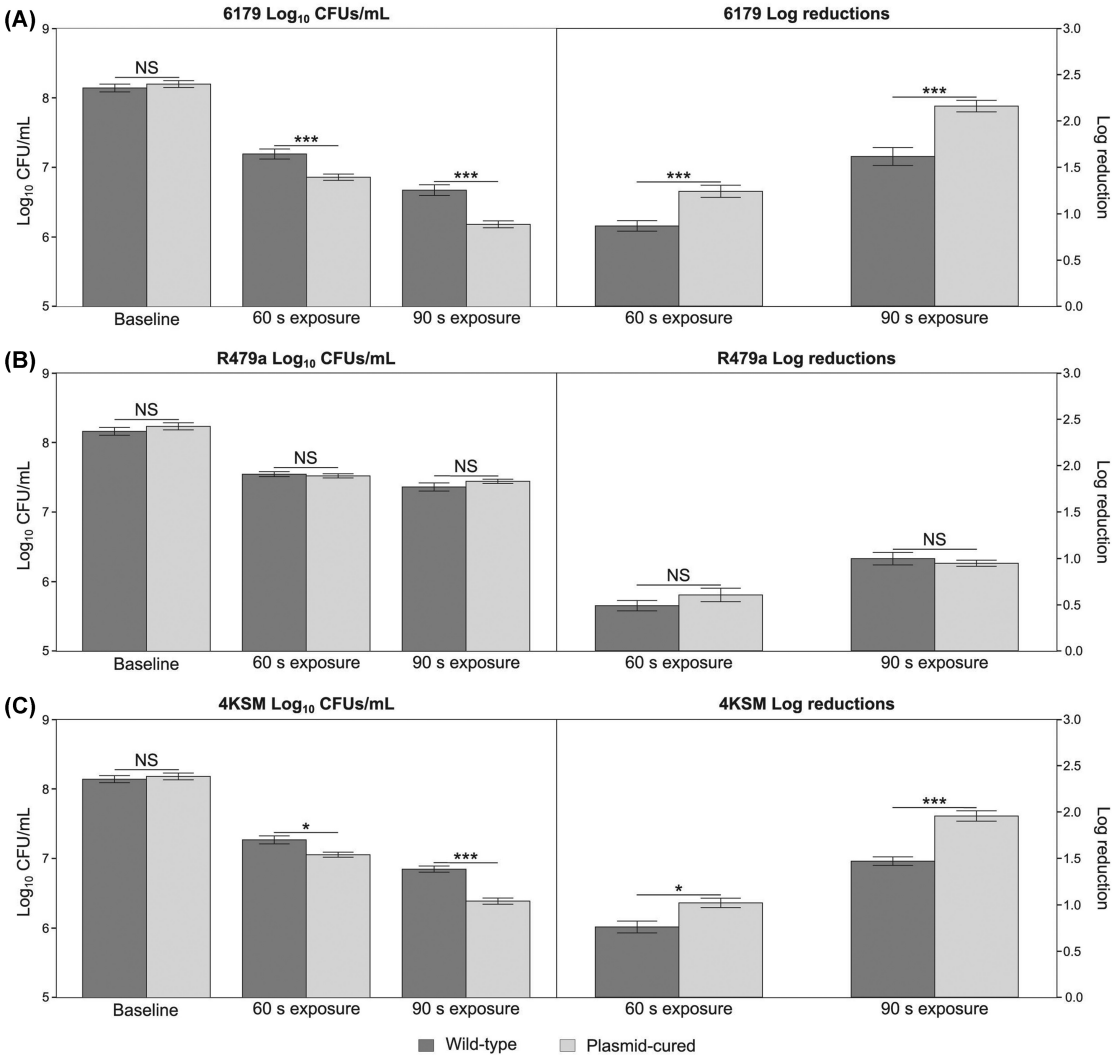
Survival of late-stationary phase WT and plasmid-cured *L. monocytogenes* strains under UVC stress at 20°C, displayed as log_10_ CFU/mL values (left panel) and log_10_ reduction values based on CFU/mL (right panel). Error bars show the standard error among four replicates. Strains appear as follows: **(A)** 6179; **(B)** R479a; **(C)** 4KSM. The baseline treatment refers to the standardized overnight (48 h) cultures that were never exposed to UVC. The 60 and 90 s treatments refer to the UVC-exposed cells. These cells were plated from the same standardized overnight as the baseline. *P*-values are as follows: *P ≤ 0.05, ***P ≤ 0.001; NS = not significant.

For late-stationary growth phase cells, the absence of pLM6179 from *L. monocytogenes* 6179 was associated with a significantly reduced survival after 60 and 90 s (log_10_ CFU and log_10_ reductions *P*-value < 0.001) UVC exposure in comparison to the WT (Table 2; Fig. 2A). Similar to the logarithmic phase cells, no significant difference between the UVC survival rates of the WT and plasmid-cured R479a strains was observed (Table 2; Fig. 2B). Additionally, the presence of p4KSM was associated with a significantly higher UVC survival rate for both the 60 s (*P*-value < 0.05) and 90 s (*P*-value < 0.001) exposures when comparing both CFUs and log_10_ reductions (Table 2; Fig. 2C). Similar to cells grown to the logarithmic growth phase, for all strains, the log_10_ reduction values were higher for the 90 s UVC exposure compared with the 60 s UVC exposure. The removal of the WT plasmid resulted in decreases of *D*-values for 6179 and 4KSM, while for R479a, the *D*-values were nearly identical.

### Genetic analysis of candidate plasmid genes for UV response

A detailed comparative genetic analysis of the plasmids pLM6179, pLMR479a and p4KSM was conducted in a previous study by our lab (Naditz *et al*. 2019); therefore, results reported here will focus on genes that may be involved with the UV stress response. After assessing the plasmid gene annotations, one putative gene was identified with a predicted function involved with the UV stress response. This putative *uvrX* gene, possibly involved in repairing DNA damage after UV stress, was present on all three plasmids. However, the length and encoded amino acid sequences of the *uvrX* gene differ between the three plasmids studied. The pLMR479a *uvrX* is predicted to encode a product 450 amino acids in length, while the pLM6179 and p4KSM *uvrX* encode shorter, 426 amino acid products. While the aforementioned predicted pLM6179 and p4KSM UvrX protein sequences are the same lengths, they are not identical and share 95% amino acid identity. Finally, the pLMR479a UvrX shares 96% and 94% amino acid identity with the pLM6179 and p4KSM UvrX proteins, respectively. Each of the three plasmid-encoded predicted UvrX protein sequences harbor three protein domains predicted to be involved in UV stress response (Pfam families: PF00817; PF11799; PF11798).

The fact that *uvrX* is present on all three plasmids but pLMR479a was not associated with a higher level of UVC survival raises the possibility that *uvrX* may not actually contribute to UVC survival. Thus, to uncover other candidate genes that may contribute to UV tolerance, we analyzed protein BLAST results of pLM6179 and p4KSM for highly similar putative proteins present on both plasmids but without homologs present on pLMR479a. One candidate gene was identified to be shared only between pLM6179 and p4KSM and is annotated as a conserved protein of unknown function (locus_tags: pLM6179, *LM6179_RS15375*; p4KSM, *UQ65_15075*) with a length of 110 amino acids (12.7% lysine residues) and an isoelectric point (p*I*) of 9.14. LM6179_RS15375 and UQ65_15075 share 100% amino acid identity. No functional domains, transmembrane helices or signal peptides have been predicted in these putative proteins, and additional homologs are only found in other *L. monocytogenes* strains. However, a distant homolog was found on the *Bacillus* pXO2 plasmid (GBAA_pXO2_0041, 30% amino acid identity and 97% coverage). GBAA_pXO2_0041 is 128 amino acids in length and has a predicted p*I* of 8.5 and a high percentage of lysine residues (16%), as well as harboring no putative functional domains, transmembrane helices or signal peptides.

## DISCUSSION

*Listeria monocytogenes* is of great concern to food producers because of its long-term persistence in food production facilities despite multiple stressors found in these environments (Ferreira *et al*. 2014; Bucur *et al*. 2018). *Listeria monocytogenes* is routinely exposed to various wavelengths of UV light throughout its saprophytic life cycle, facing UV stress both naturally on vegetation and soil and in FPEs from UV lamps meant to sanitize the facility. Exposure to UV may lead to DNA damage and the subsequent death of the cell (Rastogi *et al*. 2010). Increased UVC tolerance has been described in *L. monocytogenes*, and some chromosomally encoded genetic mechanisms for UV tolerance have been characterized (Kim *et al*. 2006; Ozer and Demirci 2006; Keklik, Demirci and Puri 2009; Bernbom, Vogel and Gram 2011; Gayan *et al*. 2015). Interestingly, UVC stress adaptation is attributed to plasmids in bacterial species other than *L. monocytogenes* (Cazorla *et al*. 2008). However, no data are available on the functional characteristics of *L. monocytogenes* plasmids in reference to increased UV stress survival. Therefore, we investigated the survival of *L. monocytogenes* WT and plasmid-cured strains in the logarithmic or late-stationary growth phase exposed to UVC.

No differences in CFUs were observed between each strain and its plasmid-cured counterpart, demonstrating that plasmid curing did not affect cell survival under controlled nonstress conditions. Similarly, in a recent study (Naditz *et al*. 2019), we determined that after the growth of the same strains under standard culture conditions at 20°C (i.e. no added stress challenge), there was no significant difference in growth between the plasmid-cured and WT strains. Thus, the plasmid curing had no general negative effect on the growth behavior of the strains. We observed that the WT *L. monocytogenes* strains 4KSM and 6179, which possessed their native plasmids p4KSM and pLM6179, survived UVC stress at a significantly higher rate than their isogenic, plasmid-cured counterparts in both logarithmic and late-stationary growth phases and exposure times. However, we did not observe a difference in phenotype between R479a and the plasmid-cured derivative after exposure to UVC stress in either the growth phase or treatment. The absence of a survival phenotype between R479a and R479a-cured strains suggests that pLMR479a does not contribute toward UVC stress tolerance, at least in the conditions tested here.

Generally, WT *D*-values were numerically higher than the *D*-values of their respective plasmid-cured strain (except for the logarithmic phase R479a where the *D*-value of the plasmid-cured strain was slightly higher), indicating that the plasmids pLM6179 and p4KSM increase the exposure time needed to reduce bacterial numbers by one log. Interestingly, the stationary phase *D*-values for all strains numerically increased compared with their respective logarithmic phase *D*-values, suggesting that the physiological state of the stationary phase better primes cells for survival against UVC exposure. However, in another study assessing *L. monocytogenes* and environmental and biological influence on UVC resistance, the authors found no significant difference in UV resistance between logarithmic and stationary phase cells (Gayan *et al*. 2015). Thus, our findings demonstrate for the first time that the plasmids of certain *L. monocytogenes* strains contribute toward increased survival during UVC stress exposure. In addition, our results show that increased UVC stress tolerance conferred by *L. monocytogenes* plasmids is plasmid specific. We are confident that the results obtained here are due to the presence or absence of plasmids for the following reasons: The three plasmids tested in this study are genetically distinct, thereby providing evidence why pLM6179 and p4KSM showed different results than pLMR479a. In addition, all tests were performed with isogenic strain pairs that differed only with respect to plasmid presence or absence. Furthermore, the observation that the baseline CFUs were not significantly different between WT and plasmid-cured strains before UV exposure also shows that the observed effects on UVC survival are due to the plasmids.

Initially, we were prompted to test the UV stress survival contribution of pLM6179, pLMR479a and p4KSM because (among other supporting evidence mentioned in the ‘Introduction’ section) a predicted *uvrX* gene was annotated on all three plasmids. The absence of a survival phenotype in R479a was interesting because we had anticipated an effect to be observed due to pLMR479a harboring a putative *uvrX* gene highly similar to the *uvrX* genes of pLM6179 and p4KSM. However, the pLMR479a *uvrX* is not genetically identical to the pLM6179 and p4KSM *uvrX* and, therefore, may function in a separate biological process or may be active in UVC stress under conditions not tested here. The aforementioned observations and the fact that *L. monocytogenes* plasmids are highly conserved in a modular fashion (Schmitz-Esser, Anast and Cortes 2021) led us to search for other candidate genes on the pLM6179 and p4KSM plasmids, which might explain why they conferred an increased survival rate. BLASTp analysis revealed a single candidate gene (locus_tags: *LM6179_RS15375/UQ65_15075*) shared between pLM6179 and p4KSM that is annotated as encoding a conserved protein of unknown function. Interestingly, *LM6179_RS15375/UQ65_15075* flanks the putative *uvrX* on the opposite strand in both pLM6179 and p4KSM plasmids, respectively. We observed that LM6179_RS15375/UQ65_15075 has a high concentration of lysine residues and an p*I* of 9.1, which suggests that the protein may interact with nucleic acids (García-García and Draper 2003; Yao *et al*. 2016): This is in line with other UV repair systems that alleviate UV-induced DNA damage (Crowley *et al*. 2006; Kim *et al*. 2006). Thus, in addition to the putative *uvrX* of pLM6179 and p4KSM, the predicted *LM6179_RS15375/UQ65_15075* may be a candidate gene involved in the plasmid-mediated UVC stress response. However, we cannot rule out that pLM6179 and p4KSM may have distinct mechanisms that confer UVC resistance in addition to or instead of the *uvrX* and *LM6179_RS15375/UQ65_15075* genes.

This investigation analyzed the native plasmids of three *L. monocytogenes* strains of diverse sequence types and their potential roles in UVC stress survival. To the best of our knowledge, our study provides the first evidence that certain *L. monocytogenes* plasmids confer increased UV stress tolerance. Furthermore, these data provide additional knowledge on the contribution of *L. monocytogenes* plasmids in FPE-relevant stress conditions. In addition, we have identified a yet uncharacterized plasmid-encoded gene, *LM6179_RS15375/UQ65_15075*, which may be involved in plasmid-conferred UVC stress tolerance. Future studies will be needed to determine whether *LM6179_RS15375/UQ65_15075* or UvrX is indeed involved in UV stress response. Currently, there is no genetic system to manipulate large *Listeria* plasmids such as pLM6179 and p4KSM; thus, generating deletion mutants of the UV candidate genes was out of the scope of this study. Additional evidence for a role of these plasmids in UVC stress response could derive from future studies that could try to transform those plasmids into other strains—either *L. monocytogenes* or different bacteria such as *Escherichia coli* or *Bacillus*—to determine whether the phenotype of interest is also observed in other hosts. More generally, plasmids of *L. monocytogenes* are highly conserved, and therefore, results pertaining to the UVC survival of strains used here may be applicable to other *L. monocytogenes* strains. Future work should build upon our data to uncover the specific genes and mechanism(s) conferred by *L. monocytogenes* plasmids that may aid in persistence despite microbial control mechanisms such as UVC light used in food production facilities.

## ACKNOWLEDGMENTS

We would like to extend our gratitude to Bienvenido Cortes for his extensive feedback during the development of this manuscript and to Lucille Jonas and Scott Starr for their contributions to data collection.
